# Use of influenza antivirals in patients hospitalized in Hong Kong, 2000-2015

**DOI:** 10.1371/journal.pone.0190306

**Published:** 2018-01-19

**Authors:** Benjamin J. Cowling, Celine S. L. Chui, Wey Wen Lim, Peng Wu, Christopher K. M. Hui, J. S. Malik Peiris, Esther W. Chan

**Affiliations:** 1 WHO Collaborating Centre for Infectious Disease Epidemiology and Control, School of Public Health, Li Ka Shing Faculty of Medicine, The University of Hong Kong, Hong Kong Special Administrative Region, China; 2 Department of Medicine, Li Ka Shing Faculty of Medicine, The University of Hong Kong, Hong Kong Special Administrative Region, China; 3 Centre of Influenza Research, Li Ka Shing Faculty of Medicine, The University of Hong Kong, Hong Kong Special Administrative Region, China; 4 Centre for Safe Medication Practice and Research, Department of Pharmacology and Pharmacy, Li Ka Shing Faculty of Medicine, The University of Hong Kong, Hong Kong SAR, China; The University of Chicago, UNITED STATES

## Abstract

**Objectives:**

We aimed to describe patterns in the usage of antivirals to treat influenza virus infection in hospitals in Hong Kong from 2000 through 2015.

**Methods:**

We analyzed centralized electronic health records that included dispensation information and diagnosis codes. Information collected on admissions included patient age, sex, admission year and month, and medications dispensed, and were matched with the first 15 discharge diagnosis codes. We divided monthly admission episodes by relevant population denominators to obtain admission rates, and stratified analyses by drug type, age group, and diagnosis codes.

**Results:**

Amantadine was used for influenza treatment in the early 2000s but changed with recommendations to avoid its use in 2006, and is now mainly used to treat Parkinson’s disease. Oseltamivir usage increased substantially in 2009 and is now commonly used, with almost 40,000 hospitalizations treated with oseltamivir in the years 2012 through 2015, 66% of which was in persons ≥65 years of age. During the entire study period, of the 98,253 admission episodes in which oseltamivir was dispensed, 40,698 (41%) included a diagnosis code for influenza, and 80,283 (82%) included any diagnosis code for respiratory illness.

**Conclusions:**

The amount of oseltamivir used from 2012–15 was comparable to a separate ecological estimate of around 13,000 influenza-associated hospitalizations per year on average. We did not have access to individual patient laboratory testing data.

## Introduction

Influenza virus infections cause considerable morbidity and mortality each year. A number of antiviral drugs can be used to treat influenza virus infections [1]. The M2 inhibitors amantadine and rimantadine that have been used since the 1960s and 1990s respectively, although neither are currently recommended for use against influenza because of resistance in the currently circulating strains [1, 2]. The neuraminidase inhibitors oseltamivir and zanamivir were approved by the US Food and Drug Administration in 1999, and a third neuraminidase inhibitor peramivir was approved in 2014 although it had been available during the 2009 pandemic with an emergency use authorization. Peramivir and rimantadine are not registered in Hong Kong. Oseltamivir, the most widely used anti-influenza drug globally, has proven efficacy in reducing the duration of uncomplicated influenza illness in randomized controlled trials [3], and observational studies have indicated a benefit in severe influenza [3].

Hong Kong is a highly developed subtropical city located in southern China, with a population of 7.3 million in 2016. Hong Kong has a mixed healthcare system, in which approximately 73.7% of admissions occur in public sector hospitals administered by the Hospital Authority [4], and the remainder occur in private hospitals. The Hospital Authority maintains centralized electronic health records that include basic demographic information, summaries of all encounters with healthcare providers, diagnoses, procedures and medications, adverse reactions and allergies, and laboratory results.

The objective of our study was to describe the use of influenza antiviral drugs in patients admitted to public hospitals in Hong Kong, and examine how the patterns in usage compared with influenza activity over the same period.

## Methods

We obtained anonymized data from the Hospital Authority on all hospital admissions between 2000 and 2015 in which antimicrobial drugs were dispensed. Information collected on admissions included patient age, sex, admission year and month, and medications dispensed, and were matched with the first 15 discharge diagnosis codes using the first three digits of the International Classification of Diseases (ICD) 9^th^ edition. For this analysis we identified admitted patients who were dispensed any of the five influenza antiviral drugs: amantadine, oseltamivir, peramivir, rimantadine, and zanamivir. We determined whether each of these admission episodes included ICD9 diagnosis codes for influenza (487), any respiratory disease (460–519), and Parkinson’s disease (332) and multiple sclerosis (340). We obtained population denominator data by age from the Census and Statistics Department of the Hong Kong Government.

To measure influenza activity in Hong Kong, we used the same approach as in previous studies, based on sentinel surveillance data on the weekly rates of influenza-like illnesses in sentinel private general practitioners from the Centre for Health Protection, and laboratory data on the weekly rates of influenza detections by type/subtype among specimens submitted to the Public Health Laboratory Services Branch of the Centre for Health Protection [5, 6].

We divided monthly admission episodes by relevant population denominators to obtain admission rates, and stratified analyses by drug type, age group, and diagnosis codes. Our study received ethical approval from the Institutional Review Board of the University of Hong Kong.

## Results

**Fig 1:**
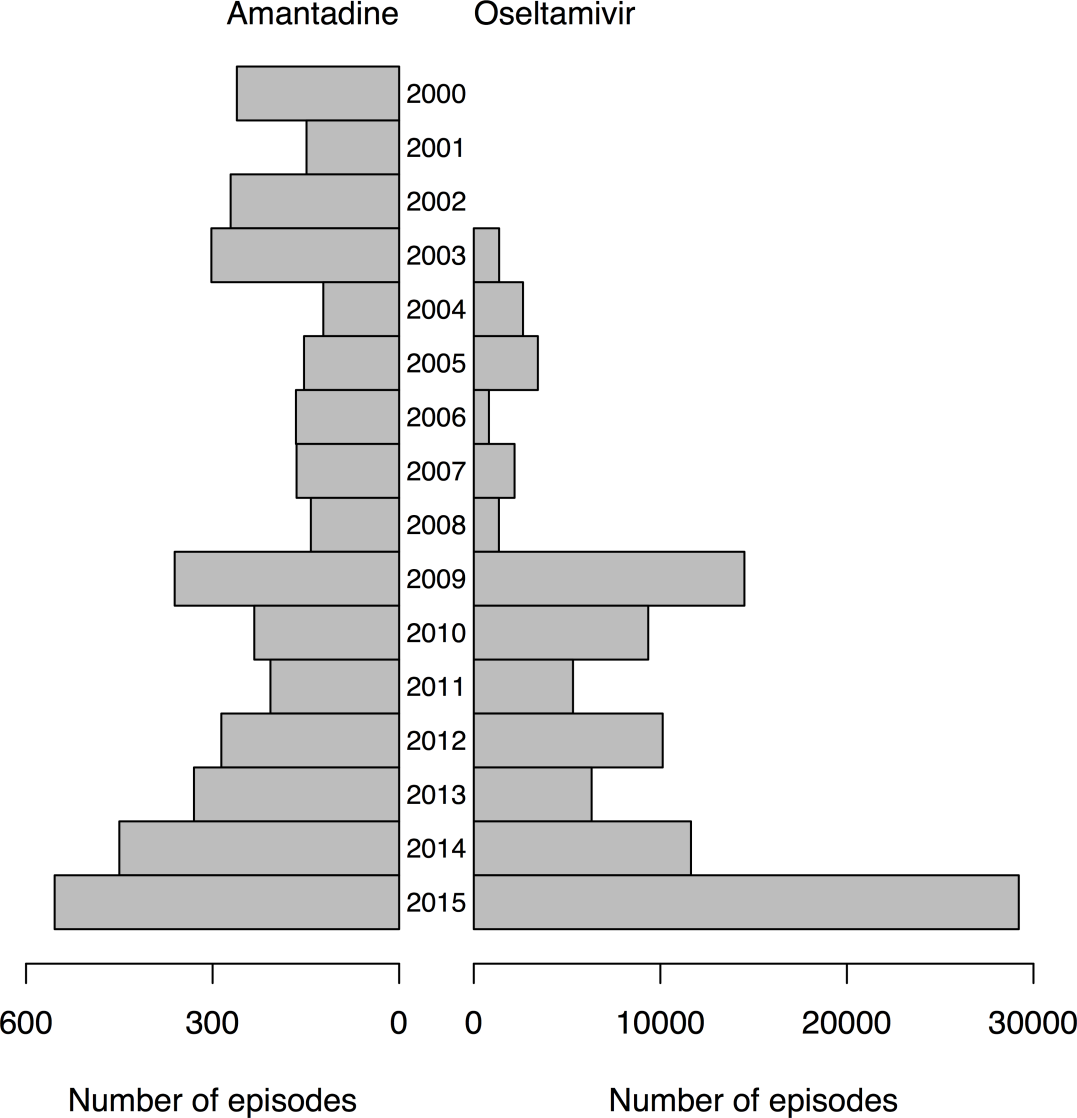
Amantadine and oseltamivir use in hospitalizations in Hong Kong, 2003–2015. The left-hand bars indicate the total number of hospitalizations in each year in which amantadine was dispensed. The right-hand bars indicate the total number of hospitalizations in each year in which oseltamivir was dispensed. Oseltamivir was licensed in Hong Kong in 2003. Note the different scales for the x-axes on the left and right side.

Of the five anti-influenza antivirals available in Hong Kong, three were registered during the study period. We identified oseltamivir dispensations to 86,477 unique patients in 98,253 admission episodes. We identified amantadine dispensations to 1,823 unique patients in 4,152 admission episodes. Fig 1 compares the annual dispensations of oseltamivir and amantadine to hospitalized patients between 2000 and 2015. We identified 939 hospitalization episodes in which zanamivir was dispensed, 90 in which peramivir was dispensed, 64/90 (71%) of which occurred in 2014 and 2015, and 4 hospitalization episodes in which rimantadine was dispensed.

**Fig 2:**
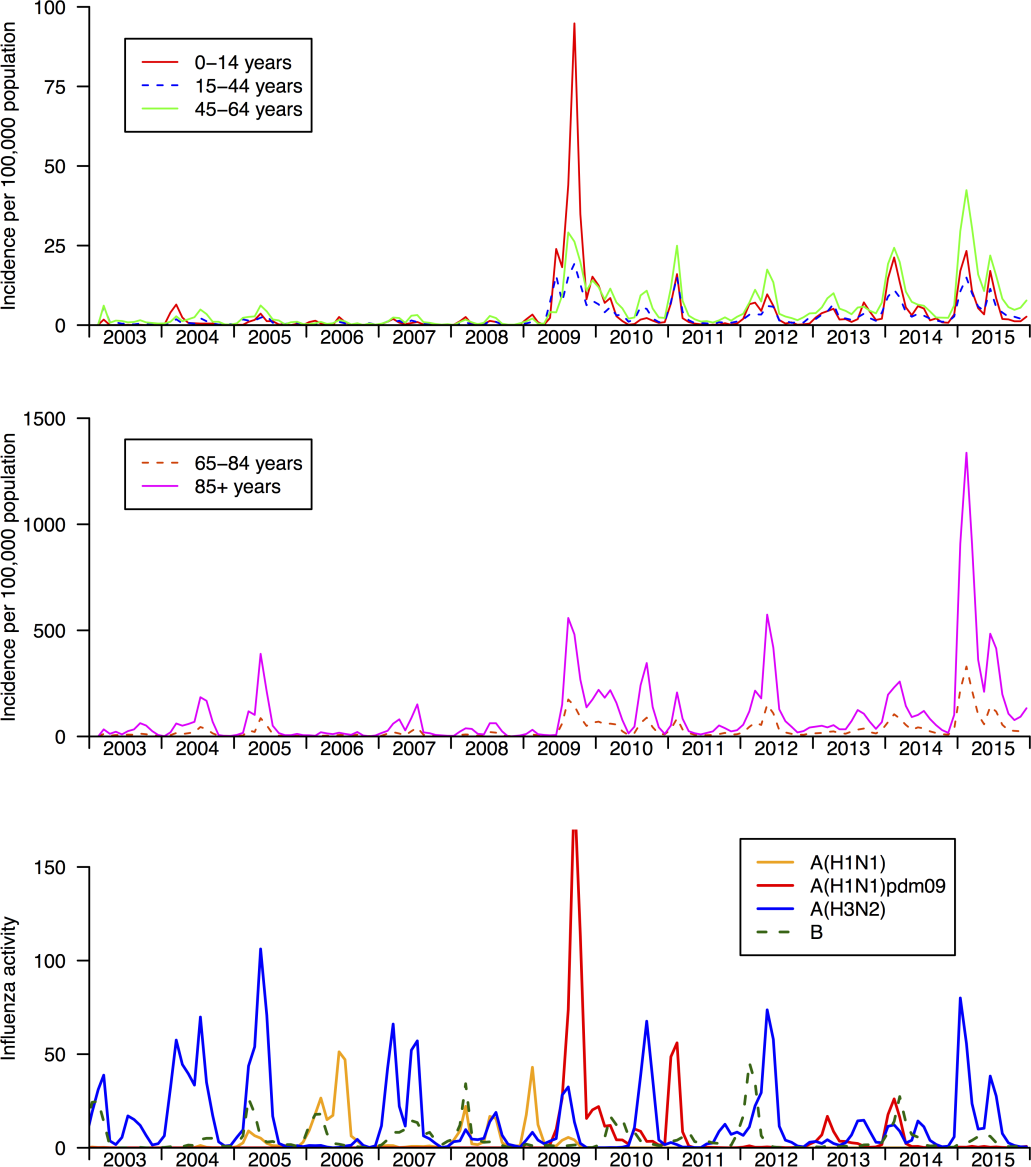
Rates of oseltamivir use in hospitalizations in Hong Kong, 2003–2015. Panel A. Rates of hospitalization in which oseltamivir was dispensed in children and adults. Panel B. Rates of hospitalization in which oseltamivir was dispensed in older adults. Panel C. Weekly influenza virus activity in Hong Kong from 1998 through 2013, measured for each influenza type/subtype as the weekly proportion of outpatient consultations associated with influenza-like-illness in sentinel outpatient clinics multiplied by the weekly proportions of laboratory specimens testing positive for influenza A(H1N1), A(H3N2), A(H1N1)pdm09 and B viruses respectively [6].

Among the 98,253 hospitalization episodes in which oseltamivir was dispensed, 66% of the episodes were in people ≥65 years of age, and 54% were males. Oseltamivir was first registered in Hong Kong in 2003. Fig 2 shows age-specific rates of hospitalization episodes in which oseltamivir was dispensed, from 2003 through 2015, where the rates were derived with population denominators, compared with influenza activity. Oseltamivir usage in admission episodes had the highest rates in persons ≥85y, with rates tracking influenza activity in all age groups. The highest rates of usage in children ≤15y occurred in the 2009/10 pandemic, peaking in September 2009 when almost 1 per 1000 children in Hong Kong were admitted to hospital and received oseltamivir. From June 2009 through April 2010, i.e. the first wave of the pandemic, oseltamivir was dispensed in 18,261 admission episodes which was 0.26% of the population of 7.0 million persons in that year.

In all age groups the rates of oseltamivir were higher in the post-pandemic period than they were before 2009, and the highest rates of usage in persons 45-64y, 65-84y, and ≥85y occurred in February 2015 at the peak of a large H3N2 epidemic with the A/Switzerland/9715293/2013 (H3N2) virus that was antigenically distinct from the H3N2 component of the 2014/15 vaccine component. In the month of February 2015, 1.3% of persons ≥85y in Hong Kong were admitted to hospital and dispensed oseltamivir. In the years 2011 through 2015, there were a total of 62,616 hospitalizations that included oseltamivir treatment, 41,591 (66%) in persons ≥65y and 2,255 (3.6%) in children ≤15y. During the entire study period, of the 98,253 admission episodes in which oseltamivir was dispensed, 40,698 (41%) included a diagnosis code for influenza, and 80,283 (82%) included any diagnosis code for respiratory illness.

**Fig 3:**
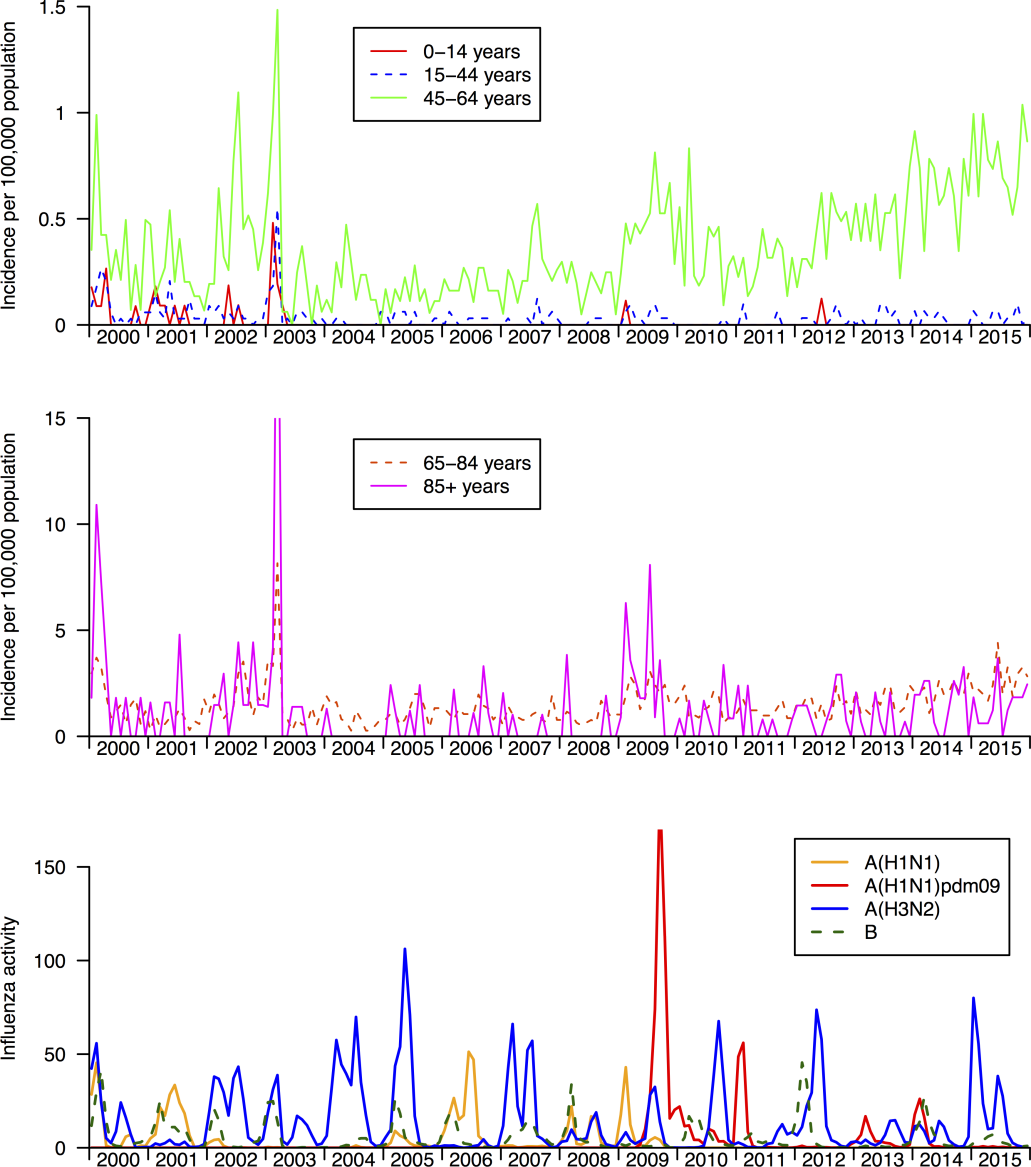
Rates of amantadine use in hospitalizations in Hong Kong, 2000–2015. Panel A. Rates of hospitalization in which amantadine was dispensed in children and adults. Panel B. Rates of hospitalization in which amantadine was dispensed in older adults. Panel C. Weekly influenza virus activity in Hong Kong from 1998 through 2013, measured for each influenza type/subtype as the weekly proportion of outpatient consultations associated with influenza-like-illness in sentinel outpatient clinics multiplied by the weekly proportions of laboratory specimens testing positive for influenza A(H1N1), A(H3N2), A(H1N1)pdm09 and B viruses, respectively [19].

Among the 4152 hospitalization episodes in which amantadine was dispensed, 60% were in patients ≥65 years of age, and 57% were males. Fig 3 shows age-specific rates of hospitalization episodes in which amantadine was dispensed, compared with influenza activity. Prior to 2003, amantadine use tracked influenza activity, shown most clearly for the 45-64y age group where multiple peaks each year match the timing of influenza peaks. Starting from 2003, when oseltamivir became available, rates of amantadine use remained relatively stable with a few notable exceptions. First, in the epidemic of Severe Acute Respiratory Syndrome (SARS) in February 2003 there were surges in amantadine use in all age groups. In the 2004/05 winter when the new A/Fujian/411/2002 (H3N2) strain had considerable impact on hospitalizations and mortality there was an increase in amantadine use in the 45-64y age group but no noticeable increase in other age groups. In the winter of 2009, an oseltamivir-resistant A(H1N1) virus circulated and amantadine use clearly increased in older adults. Another spike in usage can be seen later in the 2009–10 pandemic when the A(H1N1)pdm09 virus caused a major local epidemic. The final pattern of note is perhaps the clearest, and that is the steady rise in amantadine use in the 45-64y group since 2011, and a smaller rise in the 65-84y group over the same period. During the five years 2011–2015 there were 1827 hospitalization episodes that included an amantadine dispensation. Of these, 1037 (57%) included a diagnosis code for Parkinson’s disease and 15 (0.8%) included a diagnosis code for multiple sclerosis. Among the remainder, there were 14 (2%) with a diagnosis code for influenza and 151 (19%) with any diagnosis code for respiratory illness.

## Discussion

We studied patterns in the use of influenza antivirals in Hong Kong, finding that oseltamivir use increased considerably after the 2009–10 pandemic and in since 2009 was dispensed in approximately 10,000 hospitalization episodes per year with a notable increase in 2015 with almost 30,000 hospitalizations receiving an oseltamivir dispensation (Fig 1). In a separate ecologic analysis of admissions data from public hospitals, we estimated that over the period 1998–2013 there were an average of 12,700 influenza-associated respiratory hospitalizations per year, of which 29% were in children 0-15y and 57% of which were in older adults ≥65y [6]. From a surveillance study with comprehensive laboratory testing in two hospitals in Hong Kong, we estimated that influenza A causes an average of 1850 pediatric (<18 years) hospitalizations per year [7], while influenza B causes an average of 1200 pediatric hospitalizations in Hong Kong [8]. Our findings here suggest that oseltamivir usage approximates the estimated occurrence of influenza-associated respiratory hospitalizations in older adults, but is somewhat lower than the estimated occurrence of influenza-associated respiratory hospitalizations in children. One study in 2009 reported that almost all adults admitted with laboratory-confirmed influenza A(H1N1)pdm09 to a teaching hospital in Hong Kong received oseltamivir treatment [9]. It is interesting to note that oseltamivir dispensation to adults ≥85y was markedly greater during the influenza A(H3N2) epidemic in 2015 than during the 2009 pandemic, consistent with the much greater impact in older adults of the latter epidemic [10, 11].

There are few comparable data in the literature. Greene et al. reported population-based data on antiviral dispensing from 2000–10 based on insurance records [12]. They identified a similar drop in amantadine use for influenza from 2006 onwards, and substantial use of oseltamivir in the 2009–10 pandemic with around 180 doses dispensed per 100,000 population, 80% of which was in outpatient clinics, 18% in emergency rooms, and 2% in hospitalizations. In our study, we estimated that oseltamivir was dispensed to 0.26% of the Hong Kong population as inpatients during the 2009–10 pandemic. In a large study of more than 29,000 hospitalizations with laboratory-confirmed influenza A(H1N1pdm09) in 38 countries worldwide, Muthuri et al. reported that 60% received oseltamivir, but study sites that provided data may not have been representative of the broader health systems in those countries and the use of oseltamivir in other hospitals [3].

Given the continued resistance of circulating influenza strains to the M2 inhibitors, amantadine is mainly used to treat Parkinson’s disease since 2006, similar to the findings of Greene et al. [12], although unlike Greene et al. we did not find evidence of amantadine use for multiple sclerosis. It will be concerning if resistance to oseltamivir re-emerges on a large scale, as it did in seasonal H1N1 viruses in 2007–09 [13], because other antivirals are not available. Peramivir is being used in Hong Kong but at very low levels compared to oseltamivir, and it is not yet locally registered. However, oseltamivir and peramivir may share resistance patterns [14, 15], and it would be advantageous to increase zanamivir usage, consider licensing laninamivir, or consider other antiviral therapeutics under development.

In interpreting our findings, we note that not all oseltamivir use would be in patients with influenza virus infection, or laboratory-confirmed influenza virus infection. In some cases, it may have been used as prophylaxis, initiated in patients within 48 hours of contact with an infected individual, before laboratory results are available, or as empirical treatment of patients with influenza-like illness during periods of increased influenza activity. We found that oseltamivir usage increased considerably following the 2009–10 pandemic, and since then it appears to track patterns of influenza activity closely (Fig 2). It is not clear why oseltamivir use did not increase from 2006 onwards when amantadine was no longer recommended for use in influenza [16].

It is unclear why there was an increase in amantadine use in 2003, most notably in adults and older adults (Fig 3). To our knowledge, amantadine was not frequently used to treat patients with SARS [17]. It is possible that increased attention to pneumonia and increased laboratory testing during the SARS outbreak led to greater identification of influenza virus infections which were then treated with amantadine.

Our study has a number of limitations. First, we did not have detailed patient data and we were unable to link dispensed antivirals with symptoms and laboratory testing data, although we did match with the first 15 discharge codes. Previous work using data from the Hospital Authority has demonstrated a reasonable high coding accuracy [18], and in this study the attainment of 82% diagnostic records of respiratory illness within the first 15 discharge codes is considered reasonable. Indeed, the difficulties of the validity of diagnostic coding has been reported by others [19, 20]. Because discharge codes may not always reflect the true causes of admission, we may have misclassified the reasons for antiviral use in some patients. Second, our study only included public hospitals, which cover 73.7% of all admissions [4] and probably a greater fraction of acute admissions, but our study did not include antiviral use in outpatient settings, and therefore our estimates will be an underestimate of the total use of antivirals in Hong Kong. However, it is unlikely that we have under-ascertained antiviral use in public hospitals because prescriptions and dispensations are now managed through the electronic system that we accessed. Third, we did not examine the usage of other medications such as antipyretics or steroids in this study. Finally, we did not have sufficient information on number of doses and timing of medication, or clinical outcomes, to draw any inferences about the effectiveness of antiviral treatment in this study.

In conclusion, this study demonstrates the value of using electronic hospital records to analyze medication use, and identifies considerable oseltamivir use each year among inpatients in Hong Kong.
